# Transcriptomic evidences of local thermal adaptation for the native fish *Colossoma macropomum* (Cuvier, 1818)

**DOI:** 10.1590/1678-4685-GMB-2019-0377

**Published:** 2020-09-11

**Authors:** Luciana Mara Fé-Gonçalves, José Deney Alves Araújo, Carlos Henrique dos Anjos dos Santos, Vera Maria Fonseca de Almeida-Val

**Affiliations:** 1 Instituto Nacional de Pesquisas da Amazônia, Laboratório de Ecofisiologia e Evolução Molecular, Manaus, AM, Brazil.; 2 Universidade de São Paulo, Laboratório de Biologia de Sistema Computacional, São Paulo, SP, Brazil.

**Keywords:** Transcriptome, tambaqui, population, temperature, thermal adaptation

## Abstract

Brazil has five climatically distinct regions, with an annual average temperature difference up to 14 ºC between the northern and southern extremes. Environmental variation of this magnitude can lead to new genetic patterns among farmed fish populations. Genetically differentiated populations of tambaqui (*Colossoma macropomum* Cuvier, 1818), an important freshwater fish for Brazilian continental aquaculture, may be associated with regional adaptation. In this study, we selected tambaquis raised in two thermally distinct regions, belonging to different latitudes, to test this hypothesis. *De novo* transcriptome analysis was performed to compare the significant differences of genes expressed in the liver of juvenile tambaqui from a northern population (Balbina) and a southeastern population (Brumado). In total, 2,410 genes were differentially expressed (1,196 in Balbina and 1,214 in Brumado). Many of the genes are involved in a multitude of biological functions such as biosynthetic processes, homeostasis, biorhythm, immunity, cell signaling, ribosome biogenesis, modification of proteins, intracellular transport, structure/cytoskeleton, and catalytic activity. Enrichment analysis based on biological networks showed a different protein interaction profile for each population, whose encoding genes may play potential functions in local thermal adaptation of fish to their respective farming environments.

## Introduction

The large teleost fish, *Colossoma macropomum* Cuvier, 1818 (popularly called “tambaqui” or “cachama negra”) is a native species found in the Amazonas and Orinoco rivers (Araújo-Lima and Goulding, 1998), being economically important for Brazilian continental aquaculture (IBGE, 2016). Belonging to the Characiformes order and the Serrasalmidae family (Mirande, 2010), an adult tambaqui may reach a weight of 30 kg and a length of 1 m (Saint-Paul, 1986). Due to these traits, the tambaqui has become the primary commercial resource in Amazonian aquaculture and fisheries for its good zootechnical aspects: high level of adaptability to different culture systems, easy manipulation and reproduction in captivity by hormonal induction, high growth rate, and, of course, consumer market acceptance due to the quality of its meat (Moro *et al.*, 2013; Morais and O’Sullivan, 2017). As a result, the intensification of its production has been spread by fish farming, which is located in four distinct geographic regions of Brazil (Ostrensky *et al.*, 2008).

Brazil displays a climatic variability which can be divided into five regions; Northern, Northeastern, Central-Western, Southeastern, and Southern (Alvares *et al.*, 2013). However, the most climatically distinct Northern and Southeastern regions are highlighted in our study. According to Köppen’s classification of climates, the Northern region is naturally dominated by a humid equatorial climate (Af climate), with an annual average temperature of 27.1ºC (ranging from 22.3 to 32.6ºC), while the Southeastern region presents a humid, temperate climate (Cwa climate), with an annual average temperature of 20.1ºC (varying from 9.4 to 28.0ºC). In winter, cold fronts originating from the Atlantic polar mass may cause frost (Alvares *et al.*, 2013).

Considering seasonal temperature variation between climatic zones, recent studies have investigated the environmental adaptations of species based on genomic approaches, which reflect biological processes that are important in adaptive evolution (Yi *et al.*, 2016). Genetic variation within populations has suggested that captive tambaquis already show signs of local adaptation to regions with different climatic conditions (Santos *et al.*, 2016; Nunes *et al.*, 2017; Gonçalves *et al.*, 2019). Moreover, specific thermal adaptations of these populations have revealed differential expression of genes, displaying critical roles in metabolic processes for fish homeostasis, such as circadian rhythm, cell proliferation, energy metabolism and protein modification (Dragan, 2019).

Transcriptome analysis of non-model organisms is one of the most important approaches for providing insights into the adaptive evolution of species in response to their living environments (Yi *et al.*, 2016). However, under the current perspective of global climate change, such molecular informations may be particularly valuable in the conservation of species which are threatened by extreme environmental challenges (Bellard *et al.*, 2014). In general, fish are highly able to respond plastically to a myriad of environmental changes, but whether their plastic responses are beneficial seems to depend on the environmental variable that they are being subjected to (Schulte, 2001). Climate changes may negatively affect fish populations living close to their thermal comfort zone (Pörtner and Peck, 2010), and fish, particularly in the Amazon region, will be those most threatened (Fé-Gonçalves *et al.*, 2018; Campos *et al.*, 2019).

The genetic basis for the tambaqui fish has been developed in recent years. Thus, the present study provides a novel investigation regarding the regional adaptation of tambaqui populations raised in two thermally distinct regions of Brazil based on a comparison of transcriptome profiles.

## Material and Methods

### The historical formation of tambaqui broodstocks

The origin of farmed populations of tambaqui in Brazil dates 54 years ago. The first tambaqui broodstocks were reared between 1966 and 1970 from a few wild fish sourced from the Amazon basin (DNOCS, 2009) and the Peruvian Amazon (Araújo-Lima and Goulding, 1988). The offspring were sent to central-western, northern, northeastern, and southeastern regions to form the first local broodstocks. During the same period, adult tambaqui fish from Peruvian Amazon was taken to the UEPI (Experimental Center of Intensive Fish Farming) of DNOCS (National Department of Works for Drought Control) located in Ceará state (Araújo-Lima and Goulding, 1988). In the mid-1980s, juveniles sourced from DNOCS were also sent to other Brazilian fish farms, including Brumado Fish Farming in São Paulo state. Considering the timeline of the tambaqui breeding stock formation in Brazil, Balbina’s population has been isolated for about 50 years from the Brumado population, which is equivalent to at least 50 generations (Gonçalves *et al.*, 2019).

### Liver sampling

**Figure 1:**
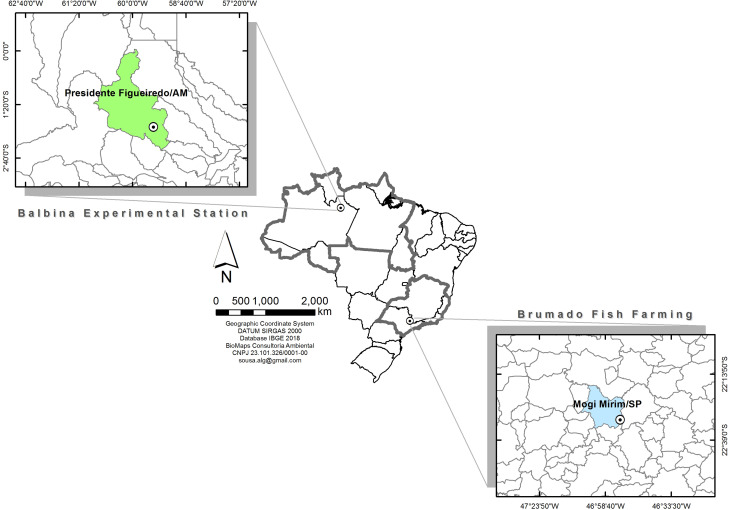
Map of the sampling sites of two tambaqui populations from different regions of Brazil. The northern (Balbina Center of Technology, Training and Production in Aquaculture, CTTPA – SEPA/SEPROR, Balbina, Amazonas state – 1°55’54.4“S; 59°24’39.1”W) and southeastern (Brumado Fish Farming, Mogi Mirim, São Paulo state – 22°31’16.00“S; 46°53’5.71”W) populations are raised in regions that display climate variability typically found in Brazil, according to Köppen’s climate classification (Alvares *et al.*, 2013).

Twenty juvenile tambaquis were collected *ex-situ* from two fish farms located in the northern and southeastern regions of Brazil (Figure 1). Sampling was carried out during the dry season when regional climate variables were similar between both sites. Thena (*n*= 10; ~ 26 g and 1 population from Balbi0 cm) was collected in June 2016, at the beginning of the Amazonian “summer” period (Fisch *et al.*, 1998), with temperatures varying between 23 to 31ºC (Climatempo, 2019). The population from Brumado (*n*= 10; ~ 60 g and 13 cm) was collected during the summer of February 2016, when temperature varied from 18.8 to 28ºC (CPTEC/INPE, 2019). At the time, the water temperature of the rearing tanks was 29.5ºC in Balbina and 21ºC in Brumado; the level of dissolved oxygen ranged from 5 to 7 mg.L^-1^.

For tissue sampling from each population, fish (42 g ± 4.7 and 11 cm ± 0.4) were anesthetized and euthanized by cervical sectioning according to Brazilian Guidelines from the National Board of Control and Care for Ethics in the use of Experimental Animals (CONCEA, 2013). Twenty liver samples were immediately stored in RNAlater® Stabilization Solution (Thermo Fisher Scientific, Massachusetts, USA) to ensure the preservation of the ribonucleic acid (RNA) during transport to the Laboratory of Ecophysiology and Molecular Evolution (LEEM/COBio/INPA), Manaus, Amazonas state, Brazil. In the laboratory, samples were removed from RNAlater®, washed in RNase-free water (Qiagen, Hilden, DE), dapped dry on an absorbent paper tissue (Whatman, GE Healthcare Life Sciences, Maidstone, UK), and then stored at -80 ºC until extraction of the RNA. Herein, the liver was analyzed tissue due to its essential metabolically responses under environmental stress (Lemgruber *et al.*, 2013; Logan and Buckley, 2015).

### Library construction for RNA sequencing

Total RNA was extracted from the tambaqui livers using RNeasy^®^ Mini Kit (Qiagen, Hilden, DE) protocol. Approximately 20 mg tissue was homogenized in lysis buffer in a TissueLyser II (Qiagen, Hilden, DE) for 2x2 minutes at 20 Hz. Automated purification of RNA was performed on a QIACube robotic workstation (Qiagen, Hilden, DE) using silica-membrane technology. The quality and quantity of extracted RNA were accurately checked using both an RNA 6000 Nano Bioanalyzer chip (Agilent Technologies, Santa Clara, USA) and a NanoDrop 2000 Spectrophotometer (Thermo Fisher Scientific, Massachusetts, USA). All the RNA samples were free of gDNA and had a suitable RNA yield (~ 0.7 μg) and optimal purity (average RIN = 9.3, A_260_:A_280_ and A_260_:A_230_ ratios = 2.0). Before library construction, three samples of total RNA were pooled, totaling six RNA-Seq libraries, with three biological replicates for each tambaqui population (Balbina and Brumado).

All procedures for constructing and sequencing of RNA-Seq libraries were carried out in the Molecular Biology Laboratory of LEEM/INPA following the Illumina protocols. The mRNA was isolated from the total RNA (0.72 μg eluted in 50 μL) using oligo d(T)25 magnetic beads bound to the poly (A) tail of the mRNA. Then, the first and second strands of complementary DNA (cDNA) were synthesized, and a single adenine (A) nucleotide was added to the end 3’ of the fragments. Adapters were ligated to the cDNA fragments and a Polymerase Chain Reaction (PCR) was performed to enrich these fragments. cDNA libraries were prepared using the reagents provided in the TruSeq RNA Library Sample Preparation Kit v2 (Illumina, San Diego, USA).

The absolute quantification of cDNA libraries was measured on a ViiA 7 Real-Time PCR System (Thermo Fisher Scientific, Massachusetts, USA) using the KAPA SYBR^®^ FAST qPCR Master Mix (Kapa Biosystems, Wilmington, USA). Normalized cDNA libraries were clustered using the MiSeq Reagent Kit v2 (500-cycles) and sequenced on an Illumina MiSeq platform in three sequencing paired-end runs (2×250 cycles). These sequence data have been submitted to the National Center for Biotechnology Information/Sequence Read Archive (NCBI/SRA) databases under accession number PRJNA547332 (https://www.ncbi.nlm.nih.gov/sra).[Bibr B54] [Bibr B55]

### Bioinformatic analysis

Analyses of the high-throughput RNA sequencing were performed at the Bioinformatics Laboratory of LEEM/INPA. The quality of sequenced reads was checked using the FastQC v.0.11.6 program (Andrews, 2010). The low-quality reads (Q-score ≤ 20) were trimmed by removing the adaptor sequences, and filtering the reads with less than 50 base pairs (bp) were performed using the Trimmomatic v.0.36 program (Bolger *et al.*, 2014). Due to the absence of the complete genome for *Colossoma macropomum* species, we choose to use the *de novo* transcriptome assembly using the Trinity v.2.5.1 program (Grabherr *et al.*, 2011). In addition, programs that assisted Trinity were used to assemble the transcriptome with the Bowtie2 v.2.3.3.1 (Langmead and Salzberg, 2012), and calculate the abundance of transcripts using the RSEM v.1.3.0 program (Li and Dewey, 2011) and R/Bioconductor packages v.3.3.2 (Bates *et al.*, 2004), respectively.

Differential expression was quantified into up- and downregulated genes using the edgeR v.3.16.5 program (Robinson *et al.*, 2009) of R/Bioconductor package. The assumed False Discovery Rate (FDR) was ≤0.05 in order to correct *P* values, and the data generated by the RSEM were used to calculate the fold change values of ≥ 2. The differentially expressed genes (DEGs) were annotated with the BLASTx v.2.7.1+ program (Altschul *et al.*, 1997), against the database of Uniprot/TrEMBL proteins (class Actinopterygii) and Swiss-Prot for non-redundant proteins, with *e*-value 1e-5. The Trinotate tool v.3.1.1 (https://trinotate.github.io/) was used to classify the DEGs according to the three general categories of Gene Ontology (GO) annotation: i) Biological Process (BP); ii) Cellular Component (CC); and iii) Molecular Function (MF).

Further analysis on Network Analyst (https://www.networkanalyst.ca/) was performed to construct relevant biological networks based on Protein-Protein Interaction (PPI) starting from a list of DEGs, using their official names and fold change values. NetworkAnalyst also allows performing functional enrichment analysis of significantly expressed GO terms according to the Kyoto Encyclopedia of Genes and Genomes (KEGG) database (Xia *et al.*, 2014).

## Results

Six cDNA libraries were constructed from the liver of juvenile tambaquis raised on the Balbina and Brumado fish farms. Three RNA-Seq runs performed on the Illumina MiSeq platform yielded 106,161,098 million (M) raw reads, with an average of 8,846,758 M reads per library. After quality trimming (Q-score < 20 and removal of reads of length < 50 bp), 100,945,530 M filtered reads were saved. About 95% of the total reads sequenced were assembled for *de novo* analysis and aligned; 166,819 contigs were assembled, and the average length was 912 bp, with an N50 value of 1,777 bp. The assembled bases totaled 152,281,627 M. Considering only those genes with a FDR < 0.05 and fold change > 2, a total of 2,410 genes showed significant differential expression between the two populations (Balbina *versus* Brumado). Of these, 1,196 (49.6%) genes were found in the Balbina population, whereas 1,214 (50.4%) genes were differentially expressed in the Brumado population. The overview of the *de novo* transcriptome statistics for the two populations of *Colossoma macropomum* is described in Table 1.

**Table 1:** Summary of the Illumina sequencing statistics.

	Balbina	Brumado
Raw reads	57,361,634	48,799,464
Min. raw reads	8,873,256	7,426,829
Max. raw reads	9,963,942	8,570,267
Average raw reads	9,560,272	8,133,244
Trimmed reads	54,363,724	46,581,806
Min. trimmed reads	8,295,129	6,990,030
Max. trimmed reads	9,465,852	8,173,557
Average trimmed reads	9,060,621	7,763,634
DE genes upregulated	622	616
Upregulated genes annotated by BLASTx	413	468
Upregulated terms assigned GO terms	3,443	4,260
DE genes downregulated	574	598
Downregulated genes annotated by BLASTx	426	389
Downregulated terms assigned GO terms	4,734	3,821

**Figure 2:**
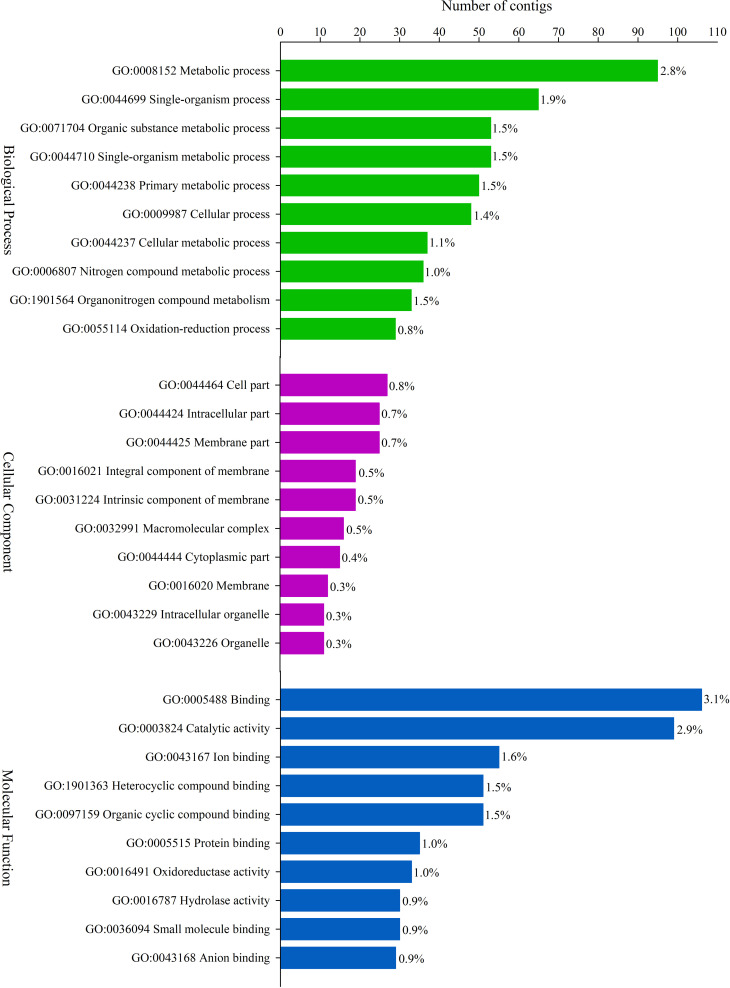
The top 30 terms classification of the contigs significantly upregulated in Balbina population and separated into three functional Gene Ontology (GO) categories: Biological Process (green bars), Cell Component (purple bars) and Molecular Function (blue bars). The percentages indicate the representation of genes that belong to each category.

**Figure 3:**
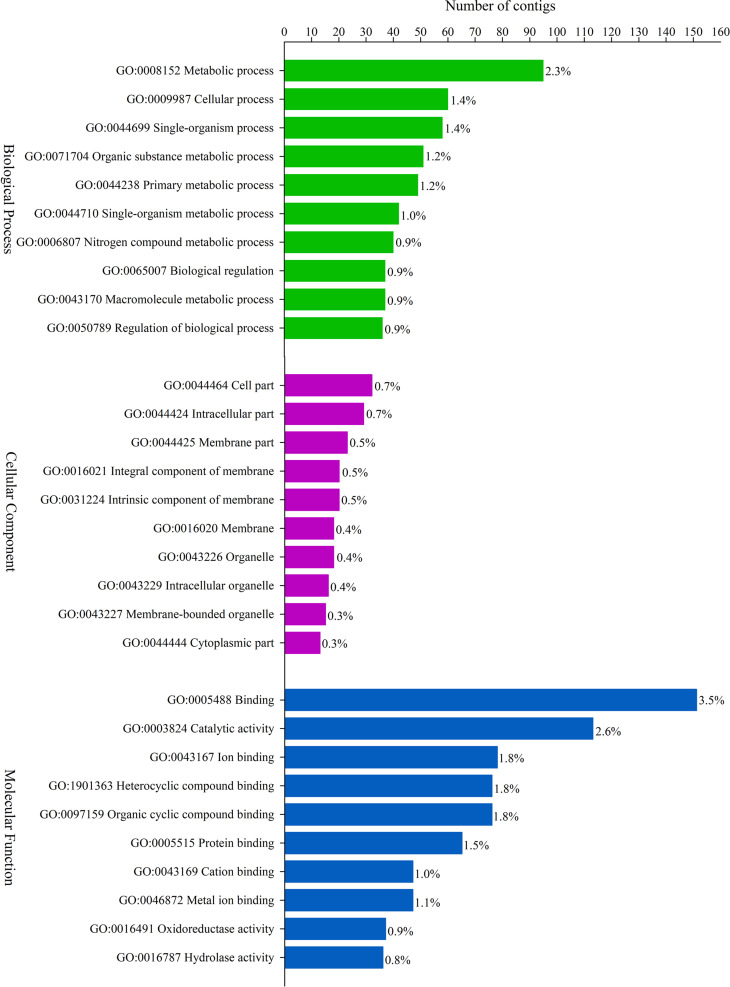
The top 30 terms classification of the contigs significantly upregulated in Brumado population and separated into three functional Gene Ontology (GO) categories: Biological Process (green bars), Cell Component (purple bars) and Molecular Function (blue bars). The percentages indicate the representation of genes that belong to each category.

Regarding the functional classification of the DEGs, only the upregulated genes were annotated through GO terms: BP – Biological Process, CC – Cell Component, and MF – Molecular Function. In the population from the Balbina farm, 3,443 terms were successfully assigned into 703 GO subcategories: BP, 1,684; CC, 318 and MF, 1,441. For the population from the Brumado farm, 4,260 terms were categorized into 851 GO subcategories: BP, 1,854; CC, 442 and MF, 1,964. GO representation of the top 30 upregulated terms identified in each population is shown in Figures 2 and 3, respectively. Forty-nine upregulated terms were shared in the two populations of tambaqui (Table 2). Overall, the genes commonly expressed between populations were related to several biosynthetic processes, homeostasis, biorhythm, immunity, cell signaling, ribosome biogenesis, metabolism of proteins, protein folding/modification, intracellular transport, structure/cytoskeleton and catalytic activity.

**Table 2:** Common terms identified between populations of tambaqui sourced from the Balbina and Brumado fish farms.

Contig ID	LogFC	Gene symbol	Gene product	GO annotation
DN15136_c0_g1_i1	12.54	IDI1	Isopentenyl-diphosphate delta isomerase 1	GO:0006629 lipid metabolic process
				GO:0016787 hydrolase activity
DN16258_c2_g1_i3	10.71	creb3l3b	Cyclic AMP-responsive element-binding protein 3-like protein 3-B	GO:0006355 regulation of transcription, DNA-templated
				GO:0001071 nucleic acid binding transcription factor activity
DN15150_c1_g1_i17	9.98	AP1G1	AP-1 complex subunit gamma-1	GO:0016192 vesicle-mediated transport
DN13446_c7_g1_i1	9.94	CNNM3	Metal transporter CNNM3	GO:0022857 transmembrane transporter activity
DN15804_c1_g1_i17	9.65	SLMAP	Sarcolemmal membrane-associated protein	GO:0008104 protein localization
DN13528_c0_g1_i2	9.11	ACIN1	Apoptotic chromatin condensation inducer 1	GO:0042981 regulation of apoptotic process
				GO:0003676 nucleic acid binding
DN13489_c2_g4_i1	8.31	Pphln1	Periphilin-1	GO:0006355 regulation of transcription, DNA-templated
DN14576_c3_g2_i5	8.30	Golt1a	Golgi transport 1A	GO:0016192 vesicle-mediated transport
DN15916_c5_g1_i3	8.28	NUFIP2	Nuclear FMR1-interacting protein 2	GO:0003723 RNA binding
DN13598_c6_g1_i5	8.18	HHIP	Hedgehog-interacting protein	GO:0009966 regulation of signal transduction
				GO:0008270 zinc ion binding
DN15473_c4_g1_i8	8.09	ADAMTSL5	ADAMTS-like protein 5	GO:1901681 sulfur compound binding
DN13646_c3_g1_i1	8.02	ECI2	Enoyl-CoA delta isomerase 2	GO:0006629 lipid metabolic process
				GO:0000062 fatty-acyl-CoA binding
DN13372_c3_g2_i5	7.92	maea	E3 ubiquitin-protein transferase MAEA	GO:0004842 ubiquitin-protein transferase activity
				GO:0070646 protein modification by small protein removal
DN12837_c0_g1_i1	7.77	MRPL9	39S ribosomal protein L9	GO:0006412 translation
				GO:0016072 rRNA metabolic process
DN16263_c2_g1_i3	7.64	PROC	Vitamin K-dependent protein C	GO:0050727 regulation of inflammatory response
				GO:0004252 serine-type endopeptidase activity
DN15506_c4_g2_i1	7.55	Cd7	T-cell antigen CD7	GO:0038023 signaling receptor activity
				GO:0016021 integral component of membrane
DN14966_c2_g1_i12	7.53	Igf2bp2	Insulin-like growth factor 2 mRNA-binding protein 2	GO:0006810 transport
				GO:0003723 RNA binding
DN15289_c7_g1_i12	7.46	KANK1	KN motif and ankyrin repeat domain-containing protein 1	GO:0006355 regulation of transcription, DNA-templated
				GO:0005515 protein binding
DN15264_c5_g2_i9	7.43	LY75	Lymphocyte antigen 75	GO:0006954 inflammatory response
				GO:0004888 transmembrane signaling receptor activity
DN15198_c0_g1_i4	7.38	LRRC41	Leucine-rich repeat-containing protein 41	GO:0070646 protein modification by small protein removal
DN14189_c1_g1_i5	7.26	CYP2J2	Cytochrome P450 2J2	GO:0006629 lipid metabolic process
				GO:0016705 cytochrome p450 activity
DN14494_c1_g2_i8	7.13	PDIA3	Protein disulfide-isomerase	GO:0006457 protein folding
				GO:0016853 isomerase activity
DN15995_c4_g1_i11	7.10	THRAP3	Thyroid hormone receptor-associated protein 3	GO:0048511 rhythmic process
				GO:0003713 transcription coactivator activity
DN14712_c2_g1_i1	7.08	NLRC3	NLR family CARD domain-containing protein 3	GO:0035556 intracellular signal transduction
				GO:0005524 ATP binding
DN15785_c1_g2_i10	6.99	glyr1	Putative oxidoreductase GLYR1	GO:0016491 oxidoreductase activity
DN15537_c3_g1_i2	6.92	MTSS1	Metastasis suppressor protein 1	GO:0007009 plasma membrane organization
				GO:0003779 actin binding
DN16282_c0_g2_i1	6.78	cgn	Cingulin	GO:0003774 motor activity
DN14613_c2_g2_i4	6.62	impad1	Inositol monophosphatase 3	GO:0016791 phosphatase activity
DN15794_c0_g1_i5	6.61	Sh3d19	SH3 domain-containing protein 19	GO:0007010 cytoskeleton organization
DN16271_c6_g1_i11	6.18	slc29a1	Solute carrier family 29member 1a	GO:0015858 nucleoside transport
				GO:0005337 nucleoside transmembrane transporter activity
DN13894_c1_g1_i10	5.48	apmap	Adipocyte plasma membrane-associated protein	GO:0009058 biosynthetic process
				GO:0016844 strictosidine synthase activity
DN13396_c1_g2_i5	5.44	cyp2k1	Cytochrome P450 2K1	GO:0030258 lipid modification
				GO:0016705 cytochrome p450 activity
DN14963_c0_g1_i20	5.35	Nlrc3	Protein NLRC3	GO:0035556 intracellular signal transduction
				GO:0005524 ATP binding
DN15981_c2_g5_i2	5.08	SUGCT	Succinate—hydroxymethylglutarate CoA-transferase	GO:0016782 transferase activity, transferring sulfur-containing groups
DN16498_c5_g1_i9	4.97	l-2	Lactose-binding lectin l-2	GO:0006952 defense response
				GO:0030246 carbohydrate binding
DN13568_c0_g2_i7	4.91	AFDN	Afadin	GO:0007155 cell adhesion
				GO:0050839 cell adhesion molecule binding
DN16570_c8_g1_i10	4.81	Cyp27a1	Sterol 26-hydroxylase	GO:0042632 cholesterol homeostasis
				GO:0004497 monooxygenase activity
DN14390_c2_g4_i1	4.77	CPT1A	Carnitine O-palmitoyltransferase 1	GO:0006629 lipid metabolic process
				GO:0016746 transferase activity, transferring acyl groups
DN16417_c2_g10_i1	4.77	CXCL8	Interleukin-8	GO:0006955 immune response
				GO:0008009 chemokine activity
DN13592_c4_g1_i17	4.59	riox1	Ribosomal oxygenase 1	GO:0016570 histone modification
				GO:0051213 dioxygenase activity
DN13897_c0_g1_i12	4.21	epd	Ependymin	GO:0007160 cell-matrix adhesion
				GO:0005509 calcium ion binding
DN16042_c2_g3_i3	4.00	Ermap	Erythroid membrane-associated protein	GO:0050776 regulation of immune response
				GO:0005102 signaling receptor binding
DN15608_c1_g1_i4	3.82	DNAJC13	DnaJ homolog subfamily C member 13	GO:0015031 protein transport
DN13357_c1_g1_i2	3.53	Tpk1	Thiamin pyrophosphokinase 1	GO:0009229 thiamine diphosphate biosynthetic process
				GO:0004788 thiamine diphosphokinase activity
DN15595_c0_g1_i1	3.41	PC	Pyruvate carboxylase	GO:0005975 carbohydrate metabolic process
				GO:0016874 ligase activity
DN13635_c1_g1_i7	3.35	AKR1B1	Aldo-keto reductase family 1 member B1	GO:0005975 carbohydrate metabolic process
				GO:0016491 oxidoreductase activity
DN16392_c0_g3_i1	3.28	PDLIM2	PDZ and LIM domain protein 2	GO:0005856 cytoskeleton
				GO:0003779 actin binding
DN15624_c0_g1_i16	3.13	Srsf5	Serine/arginine-rich splicing factor 5	GO:0006397 mRNA processing
				GO:0003723 RNA binding
DN13615_c0_g9_i2	3.06	Nop53	Ribosome biogenesis protein NOP53	GO:0006364 rRNA processing
				GO:0042802 identical protein binding

The two biological networks were constructed from the DE genes upregulated in the liver of both populations. A fully correlated seed node (or hubs) list is given in Tables S1 and S2. Each generated PPI network was composed for a suitable number of nodes (proteins) and edges (interactions between nodes); the Balbina population’s PPI presented 752 nodes and 948 edges, whereas the one of Brumado population contained 671 nodes and 818 edges.

**Figure 4:**
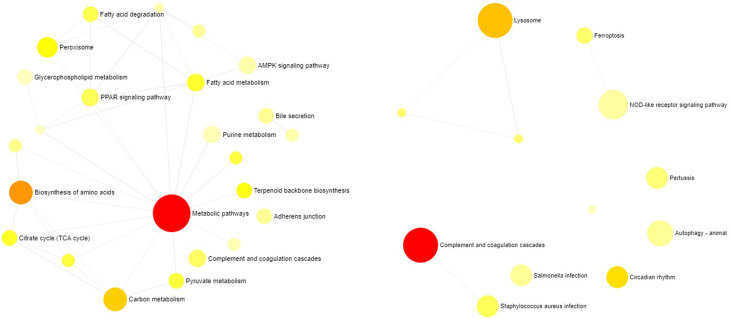
Functional representation based on KEGG pathways for differentially expressed gene-sets in the Balbina (right side) and Brumado (left side) populations.

**Figure 5:**
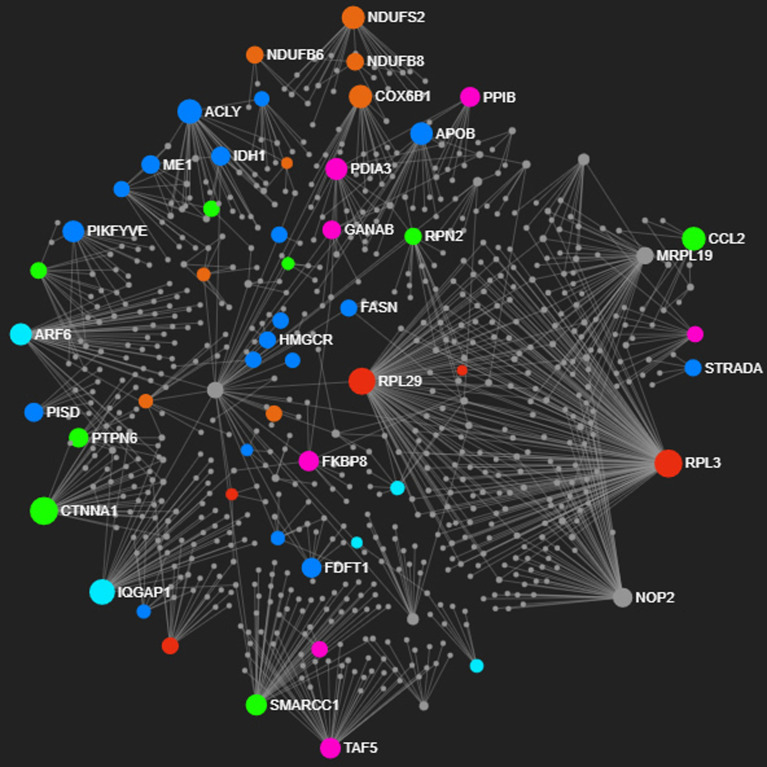
Enriched hubs highlighting the main biological processes in the protein interaction network of the Balbina population. Hubs with different colors represent prior pathways; orange – energy metabolism, dark blue – lipid metabolism, lemon green – reproductive process, light blue – RNA metabolic process, pink – protein folding, and red – intracellular protein transport. Smaller grey hubs reflect interacting non-differentially expressed genes.

**Figure 6:**
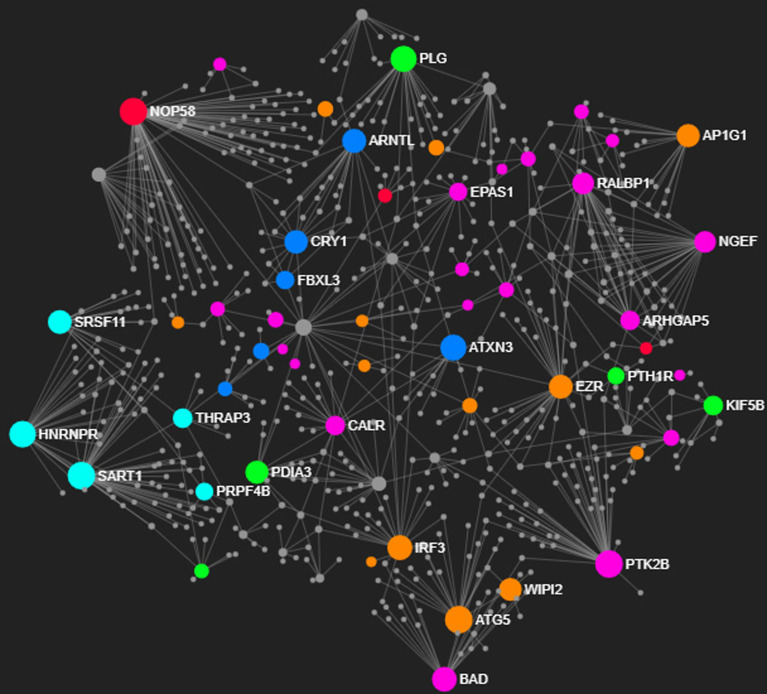
Enriched hubs highlighting the main biological processes in the protein interaction network of the Brumado population. Hubs with different colors represent prior pathways; orange – cellular response to stress, dark blue – circadian rhythm, lemon green – cellular homeostasis, light blue – mRNA processing, pink – cell signaling, and red – intracellular transport. Smaller grey hubs reflect interacting non-differentially expressed genes.

Enrichment analysis of the PPI network from each population showed a total of 36 KEGG pathways (Figure 4). Furthermore, enrichment categories based on GO terms for Biological Process were identified in both populations, as listed in Table 3. Seventy-four seed nodes were highlighted in the protein interaction network of the Balbina population (Figure 5). Proteins biologically involved in the metabolism of carbohydrates and lipids, reproduction, protein folding, and transport were represented in enriched hubs. However, the PPI network containing 70 seeds from the Brumado population showed another metabolic profile, with hub genes encoding proteins that participate in cellular homeostasis, response to external stimulus (oxygen radical, hypoxia and heat), RNA processing, signal transduction and protein import (Figure 6). Taken together, four putative functional categories involved in local adaptation of tambaqui to their respective farming sites are related to: i) energy metabolism; ii) protein folding; iii) cellular homeostasis; and iv) circadian rhythm.

**Table 3:** List of enriched biological processes represented in the protein-protein interactions (PPI) networks of both the Balbina and Brumado populations.

BP Pathways at Balbina	# Proteins	BP Pathways at Brumado	# Proteins
Lipid metabolic process	19	Regulation of biological quality	28
Organic acid metabolic process	16	Response to stress	27
Carboxylic acid metabolic process	15	Regulation of response to stimulus	24
Cellular lipid metabolic process	14	Programmed cell death	22
Carbohydrate metabolic process	13	Apoptotic process	21
Generation of precursor metabolites and energy	13	Regulation of multicellular organismal process	20
Lipid biosynthetic process	12	Immune system process	20
Nucleotide metabolic process	12	Regulation of signal transduction	19
Response to endogenous stimulus	12	Cellular localization	19
Energy derivation by oxidation of organic compounds	11	Catabolic process	18
Response to hormone stimulus	10	Cellular catabolic process	17
Coenzyme metabolic process	9	Establishment of localization in cell	17
Cofactor metabolic process	9	Regulation of apoptotic process	16
Purine nucleotide metabolic process	9	Regulation of programmed cell death	16
Alcohol metabolic process	8	Cellular component assembly	14
Intracellular protein transport	8	Intracellular transport	13
Peptidyl_amino acid modification	8	Response to external stimulus	13
Purine ribonucleotide metabolic process	8	Cellular response to stress	13
Ribonucleotide metabolic process	8	Tissue development	13
Cellular amino acid metabolic process	7	Homeostatic process	12
Cellular respiration	7	Cell migration	11
Monocarboxylic acid metabolic process	7	Enzyme linked receptor protein signaling pathway	10
Regulation of body fluid levels	7	Regulation of immune system process	10
Steroid metabolic process	7	Carbohydrate metabolic process	10
Coenzyme biosynthetic process	6	Hemopoiesis	9
Cofactor biosynthetic process	6	Hematopoietic or lymphoid organ development	9
Leukocyte migration	6	Immune system development	9
Protein folding	6	Purine ribonucleotide metabolic process	9
Steroid biosynthetic process	6	Ribonucleotide metabolic process	9
Aging	5	Purine nucleotide metabolic process	9
Glucose metabolic process	5	Cellular homeostasis	9
Negative regulation of phosphate metabolic process	5	Nucleotide metabolic process	9
Nucleotide biosynthetic process	5	Negative regulation of multicellular organismal process	8
Protein oligomerization	5	Regulation of cell migration	8
Carbohydrate biosynthetic process	4	Regulation of catabolic process	8
Cellular modified amino acid metabolic process	4	Regulation of body fluid levels	8
Energy reserve metabolic process	4	Positive regulation of immune system process	8
Isoprenoid metabolic process	4	Cell_substrate adhesion	7
Regulation of lipid metabolic process	4	Regulation of cell adhesion	7
Response to steroid hormone stimulus	4	Regulation of small GTPase mediated signal transduction	7
Triglyceride metabolic process	4	Regulation of response to external stimulus	7
Glutamine family amino acid metabolic process	3	Blood coagulation	7
Leukocyte chemotaxis	3	Coagulation	7
Protein N_linked glycosylation	3	Hemostasis	7
Protein targeting to membrane	3	Behavior	7
Response to carbohydrate stimulus	3	Vasculature development	7
Response to toxin	3	Wound healing	7
Aerobic respiration	2	Regulation of anatomical structure morphogenesis	7
Cellular modified amino acid biosynthetic process	2	Tissue remodeling	6
Excretion	2	Regulation of Rho protein signal transduction	6
		Positive regulation of cell migration	6
		Intracellular receptor mediated signaling pathway	6
		Regulation of Ras protein signal transduction	6
		Cellular component disassembly	6
		RNA splicing	6
		Regulation of cell morphogenesis	6
		Response to drug	6
		Muscle cell differentiation	6
		Nucleocytoplasmic transport	6
		Nuclear transport	6
		Leukocyte differentiation	6
		RNA splicing	6
		Positive regulation of hydrolase activity	6
		Actin cytoskeleton organization	6
		MRNA processing	6
		Positive regulation of cellular component organization	6
		Actin filament_based process	6
		Positive regulation of cell adhesion	5
		Rhythmic process	5
		Response to hypoxia	5
		Myeloid cell differentiation	5
		Leukocyte migration	5
		Cellular protein complex assembly	5
		Circadian rhythm	4
		Intracellular steroid hormone receptor signaling pathway	4
		Rho protein signal transduction	4
		Intrinsic apoptotic signaling pathway	4
		Response to carbohydrate stimulus	4
		Protein maturation	4
		Maintenance of location	4
		Post_translational protein modification	4
		Transforming growth factor beta receptor signaling pathway	4
		Protein import into nucleus	4
		Nuclear import	4
		Protein folding	4
		Extracellular structure organization	4
		Regulation of GTPase activity	4
		Lymphocyte differentiation	4
		Apoptotic signaling pathway	4
		Protein import	4
		Regulation of MAP kinase activity	4
		Ras protein signal transduction	4
		Myoblast differentiation	3
		Androgen receptor signaling pathway	3
		Regulation of JUN kinase activity	3
		B cell differentiation	3
		Regulation of Rho GTPase activity	3
		Maintenance of protein location in cell	3
		Regulation of cell shape	3
		Regulation of transforming growth factor beta receptor signaling pathway	3
		Maintenance of location in cell	3
		Maintenance of protein location	3
		Cell maturation	3
		Protein N_linked glycosylation	3
		Protein processing	3
		Leukocyte chemotaxis	3
		Regulation of JNK cascade	3
		Cyclic nucleotide metabolic process	3
		Epidermal growth factor receptor signaling pathway	3
		Protein polymerization	3
		Regulation of Ras GTPase activity	3
		Developmental maturation	3
		Focal adhesion assembly	2
		Bone remodeling	2
		Positive regulation of JUN kinase activity	2
		Vacuole organization	2
		Regulation of cell_cell adhesion	2
		Cytoplasm organization	1

## Discussion

In order to investigate the candidate genes potentially involved in the adaptation of fishes to new or constantly changing environments, the introduction of deep-sequencing technologies has provided a revolutionary tool for the precise measurement of transcript levels (Oomen and Hutchings, 2017). In the present study, we employed an RNA sequencing approach to compare the transcriptomic profile of two populations of artificially farmed tambaqui from tropical and subtropical zones in Brazil. In total, 2,410 differentially expressed genes (1,196 in Balbina and 1,214 in Brumado) which are involved in a multitude of biological functions may assign valuable information into the particular metabolic processes of each population related to regional adaptation.

It is well known that temperature drives a physical influence on the environmental adaptation of natural fish populations which live in distinct climate regions (Schulte, 2001). Based on an RNA-seq analysis, evidence for local adaptation was identified in three loaches from different climatic zones in China (Yi *et al.*, 2016). In these species of *Misgurnus*, population-specific adaptations were linked to 59 candidate genes playing functions in energy metabolism, signal transduction, membrane, and cell proliferation or apoptosis. Furthermore, comparative transcriptome-wide investigations associated to the adaptation to different environmental regimes were reported in sympatric sister species of cichlid fish from Nicaragua, *Amphilophus astorquii* and *A. zaliosus* (Elmer *et al.*, 2010), in six catfish species from gradient latitudes in the Tibetan Plateau (Ma *et al.*, 2016) as well as in cold- adaptive responses of the Antarctic notothenioid fish, *Dissostichus mawsoni* (Chen *et al.*, 2008) and Amur carp, *Cyprinus carpio haematopterus* (Liang *et al.*, 2015) to survive freezing polar conditions.

For broodstocks reared in several farming systems, among them, the two analyzed herein, regional adaptation correlated with environmental variables were first report by Nunes (2017) when comparing the eight broodstocks of tambaqui from three different climatic regions in Brazil with high throughput method. Eighteen candidate genes under positive selection were identified through genotyping-by-sequencing (GBS) and were related to the immune system, metabolism, biorhythm, and growth. According to Nunes (2017), the climatic contrast of Brazilian region may impose selective forces on the locally adapted populations. Herein, studying juveniles of the two mentioned fish facilities, the upregulation of a set of transcripts revealed the potential genes that are directly involved in the regional adaptation of each population to their living environment. After detailed functional annotation, many genes were assigned to several overlapping pathways (energy metabolism, protein folding, cellular homeostasis, and circadian rhythm), which somewhat corroborated the results described by Nunes (2017).

As stated in the literature, genetic drift strongly influences small populations that decreased in number due to some environmental constraints (Allendorf and Luikart, 2007). Randomly selected animals to form broodstocks for raising tambaqui in farms may have indeed resulted in the loss of variation due to genetic drift. However, the survival of these broodstocks along the years and generations in such a different climate must have also resulted in adaptation to the new captivity situation once, even though losing genetic variability (Santos *et al.*, 2016; Gonçalves *et al.*, 2019), generated healthy fingerlings each reproductive cycle. To proof this affirmation, we may evoke again the work accomplished by Nunes and co-workers, addressing the GBS methodology, where high-density of single-nucleotide polymorphisms (SNPs) were found to be related to thermally adaptive genes (Nunes *et al.*, 2017; Nunes, 2017) as well as in the DEGs found in the present work. On the other hand, only genetic drift would conduct farmed fish to decrease its ability to keep the reproductive success that these parents showed during all these years. While these facts are to be considered simultaneously (genetic drift and adaptive driven genes), there is a good chance, now, to choose target genes in these two populations to commercially improve these fish to the local climate where these animals are being raised.

According to Beitinger *et al.* (2000), temperature affects virtually all fish physiology. Under thermal stress, metabolic adjustments, including lipid and carbohydrate catabolism, are modulated due to the higher metabolic demand (Wang *et al.*, 2009). Compared to Brumado, at least 14 genes assigned to energy metabolism were enriched in the Balbinas biological network (Figure 5). The overexpressed genes *APOB* and *ACLY* encode proteins that participate in the lipid metabolism, indicating this may be considered the preferential energy fuel under farming climate conditions in the northern region. Likewise, we found the *FADS2* (or *scd*) upregulated gene only in this population, which assures the fluidity and flexibility of cellular membranes by increasing the level of unsaturated fatty acids (Ntambi and Miyazaki, 2004). Remarkably, Oliveira (2014) reported that higher relative transcript levels from liver *SCD-1* of tambaqui juveniles from farm cages and streams are modulated according to the daily abiotic oscillations in their breeding environment.

Besides energy metabolism, cytoskeleton organization, growth and cell death, and molecular chaperones are the main pathways of generally detected proteins in cellular stress response (Wang *et al.*, 2009). Differentially expressed proteins in the Brumado network were associated with some aspects of the responses to external stimulus (Figure 6). Particularly, heat- (*ATXN3*) and hypoxia-responsive genes (*TXN2*, *ldha*, *BAD*, *EPAS1*, *Slc29a1*, A*GTRAP*, *PTK2B*, *rest*, and *Adam8*) were enriched in this population, suggesting that their breeding environment might periodically undergo oscillations in the abiotic parameters. Moreover, in order to maintain homeostasis under variable farming conditions, fish from Brumado expressed *PDIA3*, *KIF5B*, *PLG*, and *PTH1R* genes whose proteins are responsible for cellular homeostasis. In the Balbina population, protein folding was a biologically enriched category that might be related to protein homeostasis against environmental stress (Sherman and Goldberg, 2004). Induced expression of co-chaperones such as *FKBP3*, *FKBP8*, *SLMAP*, *PPIB*, *PDIA3*, and *GANAB* genes play an essential role in assisting the proper folding of nascent or stress-damaged proteins (Wegele *et al.*, 2001; Lee *et al.*, 2011). According to Tomalty *et al.* (2015), the upregulation of chaperones (*HSP90* and *HSP70*) and associated co-chaperone genes (*CDC37*, *AHSA1*, *FKBP4*, *CHORDC1*, *HSP5A*,and *STIP1*) was strongly related to the management of denatured protein in thermally stressed juvenile Chinook salmon (*Oncorhynchus tshawytscha*). Taken together, those enriched functional categories in each population represent a relevant picture of the phenotypic plasticity that ensures the maintenance of the homeostatic state when facing the abiotic variables of their farming sites.

Biological clocks play a crucial role in controlling the many functions of organisms, ranging from subcellular processes to behaviour. The basic feature of circadian rhythm involves transcriptional feedback loop regulation being strongly associated with environmental conditions (Prokkola and Nikinmaa, 2018). Both populations of tambaqui differentially expressed genes encoding proteins involved in the positive and negative feedback loops: *PER1* in Balbina population, and *CRY1*, *ARNTL*, *ATXN3* and *FBXL3* in Brumado (Figure 6). According to Mohawk *et al.* (2012), the expression of *PER* and *CRY* transcripts drives the generating of the circadian rhythm by repressing the activity of CLOCK-ARNTL transcription factors. Notably, the upregulation of other clock-controlling genes in Brumado suggests that the seasonal changes in photoperiod in the subtropical region govern the plasticity of the rhythmicity of this population. Indeed, differential expression of circadian clock genes in response to hypoxia and temperature were observed in a cold-adapted salmonid Arctic char (*Salvelinus alpinus*) providing new insights into rhythmic regulation in fish (Prokkola *et al.*, 2018).

Thus, the suite of genes that were differentially expressed revealed the signatures of local thermal adaptation of each fish population to their environments. For the aquaculture production, the identified candidate genes can be further applied in improvement programs for the creation of more heat-tolerant tambaqui fish in the face of forecasted global climate changes.

## Supplementary material

The following online material is available for this article:

Table S1 - List of prior hubs that formed the biological network of the Balbina population.

Table S2 - List of prior hubs that formed the biological network of the Brumado population.
