# Supplementary material for: Transcriptomic evidences of local thermal adaptation for the native fish Colossoma macropomum (Cuvier, 1818)
Source: Genet Mol Biol. 2020 Sep 11;43(3):e20190377. doi: 10.1590/1678-4685-GMB-2019-0377 (PMC7485747; doi:10.1590/1678-4685-GMB-2019-0377)
Supplement: Supplementary file 1 [file 1415-4757-GMB-43-3-e20190377-s1.pdf]

Genetics and Molecular Biology
https://doi.org/10.1590/1678-4685-GMB-2019-0377

Supplementary Material to "Transcriptomic evidences of local thermal adaptation for the native fish *Colossoma macropomum* (Cuvier, 1818)"

Table S2 - List of prior hubs that formed the biological network of the Brumado population.

Gene	logFC	Protein
SGPL1	10.66	Sphingosine-1-phosphate lyase 1
CNNM3	9.94	Metal transporter CNNM3
MTMR4	9.54	Myotubularin-related protein 4
SART1	9.47	U4/U6.U5 tri-snRNP-associated protein 1
LMTK2	9.42	Serine/threonine-protein kinase LMTK2
DDX5	9.22	Probable ATP-dependent RNA helicase DDX5
ARHGAP5	9.17	Rho GTPase-activating protein 5
AP1G1	9.12	AP-1 complex subunit gamma-1
ACIN1	9.11	Apoptotic chromatin condensation inducer in the nucleus
ZC3H11A	9.05	Zinc finger CCCH domain-containing protein 11A
CANX	9.04	Calnexin
EHMT1	8.74	Histone-lysine N-methyltransferase EHMT1
FGG	8.67	Fibrinogen gamma chain
GATAD2A	8.62	Transcriptional repressor p66-alpha
TTC31	8.39	Tetratricopeptide repeat protein 31
KIF5B	8.31	Kinesin-1 heavy chain
VDAC2	8.22	Voltage-dependent anion-selective channel protein 2
PEG10	8.21	Retrotransposon-derived protein PEG10
ATG5	7.97	Autophagy protein 5
SRSF11	7.64	Serine/arginine-rich splicing factor 11
RBM19	7.57	Probable RNA-binding protein 19
WIPI2	7.47	WD repeat domain phosphoinositide-interacting protein 2
PAQR3	7.28	Progestin and adipoQ receptor family member 3
PLG	7.26	Plasminogen
SLC29A1	7.20	Equilibrative nucleoside transporter 1
ATXN3	7.18	Ataxin-3
PDIA3	7.13	Protein disulfide-isomerase A3
GNG7	7.11	Guanine nucleotide-binding protein G(I)/G(S)/G(O) subunit gamma-7
THRAP3	7.10	Thyroid hormone receptor-associated protein 3
CYB5R1	7.07	NADH-cytochrome b5 reductase 1
KLC4	7.02	Kinesin light chain 4
SPATA13	6.93	Spermatogenesis-associated protein 13
BAD	6.89	Bcl2-associated agonist of cell death
TIAL1	6.89	Nucleolysin TIAR
EPAS1	6.84	Endothelial PAS domain-containing protein 1
HNRNPR	6.77	Heterogeneous nuclear ribonucleoprotein R
IRF3	6.68	Interferon regulatory factor 3
SLC3A2	6.66	4F2 cell-surface antigen heavy chain
NOP58	6.63	Nucleolar protein 58
PRPF4B	6.41	Serine/threonine-protein kinase PRP4 homolog
MAGI1	6.30	Membrane-associated guanylate kinase, WW and PDZ domain-containing protein 1
DAB2	6.24	Disabled homolog 2
APOH	6.23	Beta-2-glycoprotein 1
NKTR	6.20	NK-tumor recognition protein
ARHGEF18	6.19	Rho guanine nucleotide exchange factor 18
A2M	5.80	Alpha-2-macroglobulin

MMP9	5.75	Matrix metalloproteinase-9
LGALS1	5.60	Galectin-1
MMP13	5.43	Collagenase 3
EZR	5.16	Ezrin
SAR1A	5.16	GTP-binding protein SAR1a
NGEF	5.04	Ephexin-1
CPT1A	4.77	Carnitine O-palmitoyltransferase 1, liver isoform
NFE2L1	4.74	Endoplasmic reticulum membrane sensor NFE2L1
PTK2B	4.55	Protein-tyrosine kinase 2-beta
RALBP1	4.42	RalA-binding protein 1
NLRX1	4.40	NLR Family member X1
PTPRJ	4.31	Receptor-type tyrosine-protein phosphatase eta
KLKB1	4.26	Plasma kallikrein
CRY1	4.03	Cryptochrome-1
PTH1R	4.02	Parathyroid hormone/parathyroid hormone-related peptide receptor
CBR1	3.67	Carbonyl reductase [NADPH] 1
FLNB	3.65	Filamin-B
ARNTL	3.45	Aryl hydrocarbon receptor nuclear translocator-like protein 1
AKR1B1	3.35	Aldose reductase
PDLIM2	3.28	PDZ and LIM domain protein 2
ARRDC3	3.25	Arrestin domain-containing protein 3
CALR	2.63	Calreticulin
CBS	2.49	Cystathionine beta-synthase
FBXL3	2.32	F-box/LRR-repeat protein 3
