# Supplementary material for: Transcriptomic evidences of local thermal adaptation for the native fish Colossoma macropomum (Cuvier, 1818)
Source: Genet Mol Biol. 2020 Sep 11;43(3):e20190377. doi: 10.1590/1678-4685-GMB-2019-0377 (PMC7485747; doi:10.1590/1678-4685-GMB-2019-0377)
Supplement: Supplementary file 2 [file 1415-4757-GMB-43-3-e20190377-s2.pdf]

Genetics and Molecular Biology
https://doi.org/10.1590/1678-4685-GMB-2019-0377

Supplementary Material to "Transcriptomic evidences of local thermal adaptation for the native fish *Colossoma macropomum* (Cuvier, 1818)"

Table S1 - List of prior hubs that formed the biological network of the Balbina population.

Gene	logFC	Protein
PPIB	10.91	Peptidyl-prolyl cis-trans isomerase B
KCNMA1	10.81	Calcium-activated potassium channel subunit alpha-1
PDIA3	10.71	Protein disulfide-isomerase A3
SMARCC1	10.21	SWI/SNF complex subunit SMARCC1
PNP	10.21	Purine nucleoside phosphorylase
MRPL9	9.98	39S ribosomal protein L9, mitochondrial
ALDH16A1	9.90	Aldehyde dehydrogenase family 16 member A1
ABCC4	9.79	Multidrug resistance-associated protein 4
GANAB	9.59	Neutral alpha-glucosidase AB
CTNNA1	9.53	Catenin alpha-1
CNNM3	9.50	Metal transporter CNNM3
AP1G1	8.97	AP-1 complex subunit gamma-1
PPP2R3B	8.95	Serine/threonine-protein phosphatase 2A regulatory subunit B" subunit beta
TTC31	8.85	Tetratricopeptide repeat protein 31
GOT1	8.83	Aspartate aminotransferase, cytoplasmic
HMGCR	8.65	3-hydroxy-3-methylglutaryl-coenzyme A reductase
ARF6	8.64	ADP-ribosylation factor 6
PDLIM2	8.59	PDZ and LIM domain protein 2
PIKFYVE	8.59	1-phosphatidylinositol 3-phosphate 5-kinase
SLMAP	8.73	Sarcolemmal membrane-associated protein
RIOK3	8.41	Serine/threonine-protein kinase RIO3
TFPI	8.40	Tissue fator pathway inhibitor
EDNRB	8.27	Endothelin receptor type B
APOB	8.25	Apolipoprotein B-100
IQGAP1	8.21	Ras GTPase-activating-like protein IQGAP1
ADSS	8.19	Adenylosuccinate synthetase isozyme 2
MTSS1	8.09	Metastasis supressor protein 1
ACIN1	8.09	Apoptotic chromatin condensation inducer in the nucleus
FKBP8	8.06	Peptidyl-prolyl cis-trans isomerase FKBP8
NOP2	7.71	Probable 28S rRNA (cytosine(4447)-C(5))-methyltransferase
RPL29	7.66	60S ribosomal protein L29
IDH3G	7.64	Isocitrate dehydrogenase [NAD] subunit gamma, mitochondrial
TOM1	7.58	Target of Myb protein 1
THRAP3	7.53	Thyroid hormone receptor-associated protein 3
SQLE	7.41	Squalene monooxygenase
MRPL19	7.19	39S ribosomal protein L19, mitochondrial
RPL3	7.04	60S ribosomal protein L3
STRADA	6.88	STE20-related kinase adapter protein alpha
CCL2	6.66	C-C motif chemokine 2
ME1	6.47	NADP-dependent malic enzyme
FDPS	6.36	Farnesyl pyrophosphate synthase
CCL13	6.14	C-C motif chemokine 13
RBM39	6.03	RNA-binding protein 39
PTPN6	5.37	Tyrosine-protein phosphatase non-receptor type 6
IDH1	5.18	Isocitrate dehydrogenase [NADP] cytoplasmic
FASN	5.08	Fatty acid synthase

SLC7A11	4.99	Cystine/glutamate transporter
NLRP12	4.90	NACHT, LRR and PYD domains-containing protein 12
ALDH18A1	4.84	Delta-1-pyrroline-5-carboxylate synthase
CPT1A	4.69	Carnitine O-palmitoyltransferase 1, liver isoform
AKR1B1	4.55	Aldose reductase
ACLY	4.49	ATP-citrate synthase
ASNS	4.43	Asparagine synthetase [glutamine-hydrolyzing]
FDFT1	4.00	Squalene synthase
PER1	3.95	Period circadian protein homolog 1
PC	3.70	Pyruvate carboxylase, mitochondrial
RDH12	3.66	Retinol dehydrogenase 12
FADS2	3.49	Fatty acid desaturase 2
PISD	3.42	Phosphatidyl serine decarboxylase proenzyme, mitochondrial
TKTL2	3.36	Transketolase-like protein 2
MAT2A	3.32	S-adenosylmethionine synthase isoform type-2
TKT	3.22	Transketolase
RPN2	2.94	Dolichyl-diphosphooligosaccharide--protein glycosyltransferase subunit 2
ACSS2	2.86	Acetyl-coenzyme A synthetase, cytoplasmic
NDUFB6	2.80	NADH dehydrogenase [ubiquinone] 1 beta subcomplex subunit 6
NME4	2.71	Nucleoside diphosphate kinase, mitochondrial
FKBP3	2.68	Peptidyl-prolyl cis-trans isomerase FKBP3
COX6B1	2.60	Cytochrome c oxidase subunit 6B1
HINT1	2.43	Histidine triad nucleotide-binding protein 1
ACP1	2.39	Low molecular weight phosphotyrosine protein phosphatase
TAF5	2.33	Transcription initiation factor TFIID subunit 5
NDUFB8	2.24	NADH dehydrogenase [ubiquinone] 1 beta subcomplex subunit 8, mitochondrial
NDUFS2	2.22	NADH dehydrogenase [ubiquinone] iron-sulfur protein 2, mitochondrial
ENO1	2.17	Alpha-enolase
